# Celecoxib Analogues for Cancer Treatment: An Update on OSU-03012 and 2,5-Dimethyl-Celecoxib

**DOI:** 10.3390/biom11071049

**Published:** 2021-07-16

**Authors:** Cyril Sobolewski, Noémie Legrand

**Affiliations:** 1 Department of Cell Physiology and Metabolism, Faculty of Medicine, University of Geneva, CH-1211 Geneva, Switzerland; 2 Department of Microbiology and Molecular Medicine, Faculty of Medicine, University of Geneva, CH-1211 Geneva, Switzerland; noemie.legrand@unige.ch

**Keywords:** cancer, celecoxib, OSU-03012, DMC

## Abstract

Cyclooxygenase-2 (COX-2) is an important enzyme involved in prostaglandins biosynthesis from arachidonic acid. COX-2 is frequently overexpressed in human cancers and plays a major tumor promoting function. Accordingly, many efforts have been devoted to efficiently target the catalytic site of this enzyme in cancer cells, by using COX-2 specific inhibitors such as celecoxib. However, despite their potent anti-tumor properties, the myriad of detrimental effects associated to the chronic inhibition of COX-2 in healthy tissues, has considerably limited their use in clinic. In addition, increasing evidence indicate that these anti-cancerous properties are not strictly dependent on the inhibition of the catalytic site. These findings have led to the development of non-active COX-2 inhibitors analogues aiming at preserving the antitumor effects of COX-2 inhibitors without their side effects. Among them, two celecoxib derivatives, 2,5-Dimethyl-Celecoxib and OSU-03012, have been developed and suggested for the treatment of viral (e.g., recently SARS-CoV-2), inflammatory, metabolic diseases and cancers. These molecules display stronger anti-tumor properties than celecoxib and thus may represent promising anti-cancer molecules. In this review, we discuss the impact of these two analogues on cancerous processes but also their potential for cancer treatment alone or in combination with existing approaches.

## 1. Introduction

Chronic inflammation has been recognized as a critical tumor promoter [1]. Among the inflammatory mediators involved in carcinogenesis, arachidonic acid-derived lipid mediators such as prostaglandins contribute to tumor development by altering several cancerous hallmarks [2]. Cyclooxygenases (COXs), also known as prostaglandin H synthase, is a family of myeloperoxidase proteins, involved in the biosynthesis of prostaglandins from arachidonic acid. COXs are located at the luminal side of the endoplasmic reticulum as well as in the nuclear membrane [3]. The first reaction catalyzed by this enzyme is the conversion of arachidonic acid into prostaglandin G2 (PGG_2_). The peroxidase activity converts this latest into PGH_2_ and then several isomerases convert PGH_2_ into prostaglandins D_2_, F_2_, E_2_, I_2_ and thromboxane A_2_ [4]. Prostanoids (prostaglandins, thromboxanes) are immediately released after synthesis and act locally in an autocrine and paracrine manner. Among these molecules, PGE_2_ plays an important tumorigenic function, by (i) activating extracellular receptors (e.g., EP receptors), thereby triggering intracellular signaling cascades involved in typical cancerous hallmarks (i.e., cancer cell proliferation, survival and migration) and (ii) favoring immune escape by impairing immune cells functions [2].

The cyclooxygenase family contains two members. The first is COX-1, a glycoprotein of 71kDa, which is constitutively expressed. In human, this protein is encoded by a gene on chromosome 9 and plays a major role in tissue homeostasis by regulating cellular processes like proliferation but also coagulation through thromboxane synthesis [2]. The second isoform, COX-2, is encoded by chromosome 1 and represents the inducible isoform, which is regulated by growth factors, proinflammatory cytokines (i.e., TNFα, Il-1β, Il-6) and Toll like receptors (TLRs). Accordingly, COX-2 promoter possesses an NFkB response element as well as other cytokines/growth factor response elements [5,6]. COX-2 expression is frequently induced during carcinogenesis due to the pro-inflammatory environment but also various epigenetic alterations (i.e., microRNAs: e.g., miR-101) [7]. This overexpression represents an important oncogenic/tumor promoting alteration favoring cancer cell proliferation, survival but also migration, immune escape, and angiogenesis [4]. Genetic approaches aiming at silencing COX-2 expression (e.g., siRNA) in cancer cells have uncovered various anti-tumor effects, thus firmly showing the relevance of targeting this enzyme for therapeutic purpose [8]. COX-2 displays 60% of homology with COX-1 but the major difference between the two isoforms is the presence of a side pocket in the catalytic site of COX-2, due to the substitution of the isoleucine at position 523 in COX-1 by a valine in COX-2. This side pocket is specifically targeted by a specific class of Non-Steroidal Anti-Inflammatory Drugs (NSAIDs) called COX-2 specific inhibitors. Several molecules of this class have been developed and are currently used for the treatment of chronic inflammatory diseases (e.g., rheumatoid arthritis). Moreover, these drugs have proven potent anti-tumoral or chemo preventive effects in vitro, in vivo [4] and in patients [9,10]. Some COX-2 inhibitors, such as celecoxib were even used for the prevention of familial adenomatous polyposis (FAP), which represents a major risk factor for colorectal cancer (CRC) development [10]. However, recent findings have suggested that the beneficial effects of these molecules are not completely COX-2-dependent [11,12], since genetic approaches aiming at silencing COX-2 expression does not always reproduce the effects of COX-2 specific inhibitors [13]. Moreover, the exogenous administration of prostaglandins is not always able to revert the anti-tumor properties of these molecules [12]. Finally, chronic inhibition of COX-2 activity by these molecules in healthy tissues can severely impact physiological processes (e.g., coagulation, blood pressure), thus considerably limiting their use in clinic. In light of the dissociation between the antitumor properties of these molecules and the enzymatic activity of COX-2 [14], using non-active analogs may represent a wiser approach for cancer treatment. Following this concept, several COX-2 specific inhibitors derivatives have been developed, such as celecoxib analogs, 2,5-dimethyl celecoxib and OSU-03012 (2-amino-N-[4-[5-(2-phenanthrenyl)-3-(trifluoromethyl)-1H-pyrazol-1-yl] phenyl]-acetamide). These molecules have been extensively studied in various in vitro and in vivo cancerous models and suggest important promises for cancer treatment. In this review, we summarize the current antitumor properties of these molecules, their abilities to affect typical carcinogenic processes/pathways and finally, their therapeutic potential alone or in combination with other existing therapies.

## 2. From Adverse Side Effects of COX-2 Inhibitors toward Non-Active Celecoxib Analogs

**Figure 1:**
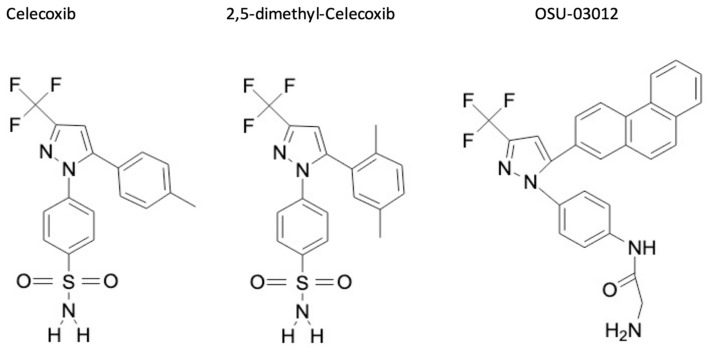
Chemical structures of celecoxib, 2,5-Dimethyl-celecoxib and OSU-03012. Chemical structures were retrieved from https://pubchem.ncbi.nlm.nih.gov (accessed on 29 April 2021) and redrawn using Chemdrawdirect (https://chemdrawdirect.perkinelmer.cloud/js/sample/index.html# accessed on 29 April 2021).

COX-2 plays an important role in various physiological processes, including vasoconstriction, protection of gastric mucosa, coagulation [15,16,17]. Therefore, numerous side effects have been associated to COX-2 inhibition by NSAIDs including gastric ulceritis, increased risk of myocardial infarction or stroke [18], thus limiting their use in patients, especially those having a cardiomyopathy history. Among the deleterious mechanisms involved, an imbalance between the metabolism of arachidonic acid sustained by COX-1 in platelets and by COX-2 in endothelial cells, which favor the production of TXA_2_ (vasoconstrictive), and reduce PGI_2_ (vasodilator), thereby fostering coagulation and vasoconstriction. Other mechanisms associated to the chronic inhibition of COX-2 may also contribute to this imbalance, including the increased LOX (lipoxygenase) pathway, which promotes the synthesis of vasoconstrictive derivatives, such as leukotriene B4 (LTB4) by neutrophils [19,20]. Due to these deleterious effects, several COX-2 inhibitors have been retrieved from the market, such as rofecoxib in 1996 [21] (https://www.fda.gov/drugs/postmarket-drug-safety-information-patients-and-providers/vioxx-rofecoxib-questions-and-answers accessed on 29 April 2021) [22]. Moreover, the other COX-2 inhibitors are deeply monitored and the question about their detrimental versus beneficial effects is still under debate (e.g., celecoxib). However, despite their side effects, these molecules possess potent anti-tumor properties and thus it has been suggested that modifying the chemical structure of COX-2 inhibitors may represent an appealing approach to preserve their anti-tumor effects and impair their binding to COX-2 catalytic domain, which are responsible for most deleterious effects. This idea has led to the birth of non-active COX-2 inhibitors analogues from celecoxib (e.g., OSU-03012, OSU-03013 and 2,5-dimethyl celecoxib) (Figure 1), which are unable to bind to COX-2 catalytic domain. Among them, OSU-03012 and 2,5-dimethyl celecoxib represent the most studied analogs and these molecules have demonstrated a great potential for the treatment of several diseases, including viral (e.g., inhibition of flavivirus replication; anti-Zika virus properties; influenza viruses, Ebola virus [23,24,25], SARS-CoV2 [26]) or bacterial infections [27] but also a variety of cancers. Moreover, these molecules can be administered orally and seem to be well tolerated in vivo and thus may represent potential therapeutic molecules for cancer treatment.

## 3. Anti-Tumor Properties of OSU-03012 and DMC

DMC and OSU03012 alter several pathways and processes involved in carcinogenesis. These molecules have demonstrated anti-tumor properties in various cancers (summarized in Table 1) and thus may represent appealing anti-cancer drugs alone or in combination with other existing approaches.

### 3.1. Effects of Non-Active COX-2 Inhibitors Analogues on Cell Cycle Progression

#### 3.1.1. 2,5-Dimethyl Celecoxib (DMC)

The growth inhibitory properties of DMC were originally found in Burkitt’s lymphoma cell lines, where DMC impairs cell cycle progression through the downregulation of cyclin-A and -B expression and the induction of the cell cycle inhibitor p27Kip1 [44]. A similar effect has been observed in leukemic cells and has been associated to the downregulation of c-myc expression, thereby impairing the G1/S transition of cell cycle [45]. Moreover, mitosis was also impaired in this study, potentially through a reduction of survivin protein expression, which belongs to the chromosomal passenger complex (CPC), involved in the proper attachment of microtubules to the kinetochore of chromosomes during mitosis [47]. Similar alterations of mitosis progression were observed in our previous study [45], showing that DMC impairs mitosis progression through a decreased survivin expression and the formation of abnormal centrosomes in leukemic cell lines [45]. Alteration of cell cycle progression by DMC was also observed in colorectal cancer in vitro (HCT-116 and DLD-1 cell lines) but also in vivo (i.e., Mutyh-/- mice, which develop multiple intestinal carcinomas due to oxidative stress in the gastric mucosa). In this study, DMC promotes the degradation of TCF7L2 (T-cell factor-like 2), which results in an impairment of the Wnt/β-catenin signaling. In Mutyh-/- mice, repeated treatment with celecoxib or DMC (respectively, 150 and 100 mg/kg orally) [28] leads to a reduction of the number of intestinal tumors, associated to a downregulation of TCF7L2, cyclin-D1 and survivin. Importantly, no toxicity was observed in these animals, thus suggesting a good tolerability for this compound. Finally, DMC impairs cell cycle progression of glioblastoma multiforme cell lines (i.e., A172, LN229, U251 and U87MG) by increasing p21 expression and suppressing CIP2A/PP2A/AKT signaling [38].

#### 3.1.2. OSU-03012 (OSU)

OSU was initially associated to an inhibition of PDK1 in prostate cancer cells (PC-3 cells), through a direct binding to its ATP binding site [33]. This effect was further confirmed in other models, including NIH/3T3 cells, where OSU prevents TCRP1 (Tongue cancer resistance-related protein 1)-induced oncogenesis, through an inhibition of PDK1 and a decreased cyclin-D1 expression [48]. Treatment of retinoblastoma cells with OSU increases the tumor suppressive function of miR-363-3p, which regulate PI3CA (phosphatidylinositol 4,5-bisphosphate 3 kinase catalytic subunit α) and PDK1 expression [49]. In human thyroid cancer cell lines, OSU inhibits AKT phosphorylation by PDK1 and leads to an accumulation of the cells in S phase of cell cycle and an induction of apoptosis [46]. However, in this study, the inhibition of PI3K with a specific inhibitor (LY294002) gave different results, thus suggesting that the effect of OSU on cell cycle involves other mechanisms. This study highlighted that OSU inhibits PAK1 (p21-Activated Kinase 1) phosphorylation, through a direct binding to the ATP-binding motif. Other PDK1-independent effects of OSU have been uncovered, such as the destabilization of MYCN and Myc proteins in neuroblastoma cells, while AKT phosphorylation is barely detectable [50]. Furthermore, in silico molecular docking analysis predicted Aurora Kinase A as a potential target of OSU with an interaction energy lower than the Aurora Kinase A specific inhibitor «FXG» [50]. OSU inhibits also directly DHOH (Dihydroorodate deshydrogenase), a key enzyme involved in pyrimidine synthesis [51]. Finally, OSU decreases the proliferation and activation of hepatic stellate cells in the liver by inducing p16, p21 and p27 protein expression. This effect not only suggests that this molecule could be of potential interest for the treatment of liver fibrosis [52] but also may prevent the progression of fibrosis toward cirrhosis and hepatocellular carcinoma.

### 3.2. Effects of OSU and DMC on Apoptosis

#### 3.2.1. Intrinsic Apoptosis

**Figure 2:**
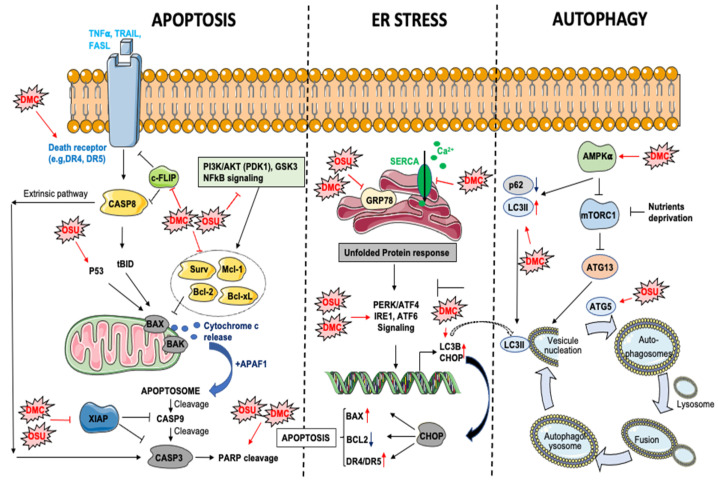
Impact of OSU-03012 (OSU) and DMC on apoptosis, ER stress and autophagy processes. AMPK: AMP-activated protein kinase. APAF1: Apoptotic peptidase activating factor 1. ATF6: Activating transcription factor 6. ATG: autophagy-related protein. BAK: Bcl-2 homologous antagonist killer. BAX: Bcl-2–associated X protein. Bcl-2: B cell lymphoma 2. Bcl-xL: B cell lymphoma extra-large. CASP: caspase. c-FLIP: cellular FLICE (FADD-like IL-1β-converting enzyme)-inhibitory protein. CHOP: C/EBP Homologous Protein. DMC: 2,5-dimethyl celecoxib. DR5: death receptor 5. ER: endoplasmic reticulum. FasL: Fas Ligand. GRP78: glucose-regulated protein 78. IRE1: Inositol-Requiring Enzyme 1. LC3: Microtubule Associated Protein 1 Light Chain 3. Mcl-1: myeloid cell leukemia 1. mTORC1: mammalian target of rapamycin complex 1. NFkB: nuclear factor kappa B. OSU: OSU-03012. PARP: poly(ADP-ribose) polymerase. PDK1: Pyruvate dehydrogenase kinase 1. PERK: protein kinase R (PKR)-like endoplasmatic reticulum kinase. PI3K: Phosphatidylinositol-3-Kinase. SERCA: sarco/endoplasmic reticulum Ca^2+^-ATPase. Surv: Survivin. TNF: Tumor necrosis factor. TRAIL: TNF-related apoptosis-inducing ligand. XIAP: X-linked inhibitor of apoptosis protein.

The inhibition of PDK1/AKT signaling by DMC or OSU treatment represents an important component of DMC/OSU-induced apoptosis in different cancers (e.g., prostate cancer cells, glioblastoma) [33,38]. However, both drugs can impair other survival pathways controlling the expression of anti-apoptotic proteins, including NFkB signaling in colon cancer [53] or phospho-GSK for OSU in breast cancer cells [29] (Figure 2). Moreover, DMC and OSU can reduce the expression of potent anti-apoptotic proteins, including BCL-2, BCL-xL Survivin, XIAP, or MCL-1 [54,55], thus triggering the intrinsic apoptotic pathway. Interestingly, the downregulation of MCL-1 is among the earliest events occurring following DMC exposure in leukemic cells [45]. OSU is also a potent apoptosis inducer in esophageal cancer cells [43] with a IC50 lower than 2 μM. This effect is mediated through the activation of the intrinsic apoptotic pathway in a p53-dependent manner. In primary chronic lymphocytic leukemia (CLL), OSU induces apoptosis in a caspases-dependent and independent manner [31] in a low micromolar range (LC50: 7.1 μM). This effect is associated with the activation of the intrinsic apoptotic pathway but independently of BCL-2 [31].

#### 3.2.2. Extrinsic Apoptosis

Several studies have demonstrated that DMC can trigger the extrinsic apoptotic pathway through different mechanisms (Figure 2), including an induction of DR5 expression in human TuBEC cells [56], thus suggesting that DMC may sensitize cancer cells to extracellular death stimuli. In agreement, Chen et al. [36] showed that DMC enhanced TRAIL (tumor-necrosis-factor related apoptosis inducing ligand)-induced apoptosis in different NSCL (non-small cell lung cancer) cancer cell lines through the upregulation of DR4 and DR5 receptors (DR: Death receptors). Furthermore, DMC decreased the expression of an inhibitor of TRAIL-induced apoptosis pathway, c-FLIP, in a proteasome-dependent manner. A similar potentiation of TRAIL-induced apoptosis by DMC, was observed in glioblastoma multiform (GBM) [37]. In this study, DMC induces ER stress and downregulates Survivin expression in both TRAIL sensitive and resistant GBM cell lines (DR5-high expressing A172 cells and DR5-low expressing U87 cells). Importantly, this effect was obtained at sub-toxic concentrations of DMC. Glioblastoma is among the deadliest cancers with few and poorly efficient therapeutic options. Downregulation of DR4 or DR5 receptor is frequently observed in GBM and contributes to TRAIL resistance. Therefore, DMC or OSU may represent efficient tools to re-sensitize GBM cancer cells to TRAIL-induced apoptosis.

#### 3.2.3. DMC and OSU-03012 Are ER Stress Aggravators

Endoplasmic reticulum-stress (ER stress) can be induced by accumulation of unfolded proteins, lipids imbalance, glucose deprivation (through alteration of protein N-glycosylation), virus infection or aberrant calcium regulation in the ER [57,58]. The first goal of the unfolded protein response (UPR) is to adapt to a new environment and restore cellular homeostasis by decreasing translation, increasing protein folding via transcriptional increase of chaperone proteins such as GRP78, and promoting endoplasmic reticulum-associated protein degradation (ERAD). In the case of a chronic ER-stress, alarm signals and danger signals can be activated, leading to cell death through apoptosis. UPR can be considered as a cellular “guardian” which can repair cellular dysfunctions or induce cell death if the alterations are too important [59]. During tumorigenesis, the rapid growth of cancer cells, together with the insufficient vascularization triggers hypoxia, glucose deprivation, oxidative stress, and errors in glycoproteins biosynthesis. However, cancer cells can maintain ER homeostasis and thus escape this physiological barrier through different mechanisms (e.g., overexpression of chaperone proteins: e.g., GRP78) [59].

DMC and OSU are potent inducers of ER-stress in various cancer cell models (Figure 2). In glioblastoma, OSU directly binds to the ATPase domain of GRP78 and inhibits its activity (ATP-competitive inhibitor) [40]. In agreement with this study, combination of OSU with sildenafil in GBM cancer cells leads to a stronger induction of ER stress associated to a decreased expression of various chaperone proteins, including GRP78, HSP70/90 proteins [60]. Furthermore, a proteomic approach allowed to identify other chaperone proteins, which directly interact with OSU, including GRP75, HSP75, BAG2, HSP27, ULK-1 and thioredoxin [60]. In glioblastoma cells, OSU reduces GRP78 protein stability and induces PERK phosphorylation and autophagy [39]. Silencing of IRE1a or ATF6 (Activating Transcription Factor 6) increases OSU-induced cell death, while the knockdown of PERK or the overexpression of a dominant negative PERK reduces its toxicity [39]. In agreement, the knockdown of IRE1 and XBP1s sensitizes parental and stem-like glioma cells to OSU-induced cell death [61]. Finally, DMC-induced ER stress has been associated to an alteration of calcium homeostasis in U251 glioblastoma cells [62]. This effect is mediated by a direct inhibition of SERCA (sarco/endoplasmic reticulum Ca^2+^ ATPase), which triggers an important leakage of calcium from the ER to the cytosol. A similar effect has also been observed in triple negative breast cancer cell lines [30].

### 3.3. DMC and OSU-03012 and Autophagy

Autophagy is a critical process involved in the recycling or the degradation of intracellular components such as macromolecules (e.g., proteins, lipids) or damaged/supernumerary organelles (e.g., mitochondria). Autophagy can be triggered under various stress conditions, such as nutrients/growth factors deprivation, oxidative stress, ER stress or hypoxia and thus represents an alternative way for the cells to maintain energy homeostasis and survival. However, in condition of prolonged stress, autophagy can also represent a cell death mechanism [63]. This process starts with the engulfment of parts of the cytoplasm within double-membraned vesicles called autophagosomes. This step is mediated by the de-repression of the mTOR kinase, which inhibits autophagy initiation by phosphorylating ATG13 protein [64]. Then, autophagosomes fuse with lysosomes to form autophagolysosomes. Alteration of autophagy has been associated to a wide spectrum of diseases including inflammatory, metabolic [65], neurological diseases [66] and cancers [67]. Moreover, the induction of autophagy in cancer cells favors chemoresistance, thus highlighting the potential of targeting this process for therapeutic purpose. DMC and OSU alter autophagy differently depending on the cancer type or the model (Figure 2). In hepatic cancer cells (i.e., Huh-7, Hep-3b and HepG2) OSU induces autophagic and reduces cell proliferation through an induction of ATG5 and an accumulation of ROS [35]. Furthermore, a reduction of tumor growth and an induction of autophagy was observed in BALB/c nude mice bearing Huh7 tumors xenografts and receiving OSU orally. The induction of autophagy by celecoxib analogs is tightly associated to the induction of ER stress, as evidenced in hepatocellular carcinoma, where DMC induces autophagy through the activation of ATF4/CHOP signaling. Moreover, DMC promotes autophagy via AMPK activation, thereby leading to LC3B-II induction and p62 downregulation [34]. In glioblastoma, the combination of OSU with an ERBB inhibitor enhances the number of autophagic vesicles in cancer cells and promotes cell death [42]. Additionally, the combination of OSU and the expression of MDA-7/IL-24 induces autophagy in GBM in an ER stress-dependent manner through PERK phosphorylation [41]. Moreover, the knockdown of IRE1 in glioblastoma cells considerably enhance OSU-induced autophagy [61]. Interestingly, although DMC and OSU can induce autophagy in several models, the inhibition of autophagy with chloroquine increases the sensitivity of triple negative breast cancer cells to DMC and nelfinavir. This effect is due to an aggravation of ER stress [30].

Together, these data further reinforce the idea that combining DMC or OSU with autophagy inhibitors may provide a better cancer cells killing. Nevertheless, autophagy is an important metabolic regulator as evidenced by ATG5KO mice, which develop hepatic steatosis and NASH (non-alcoholic steatohepatitis) [68,69,70]. These data are therefore suggesting cautions regarding the potential use of autophagy inhibitors (e.g., chloroquine, hydroxychloroquine) for the treatment of cancers. Additional studies are required to further evaluate the impact of these molecules and celecoxib analogs on the physiology of these organs.

### 3.4. Effects of OSU-03012 and DMC on Cancer Cells Migration/Invasion

The ability of OSU or DMC to affect metastasis formation was poorly studied. However, both DMC and OSU have the capacity to reduce the invasion of different cancer types through the downregulation of matrix metalloproteinases (MMPs), especially MMP-2 and 9 [71,72]. In addition, Porchia et al. demonstrated that OSU inhibits thyroid cancer cells motility (NPA, WRO and ARO cell lines) by inhibiting PDK1, but also directly inhibiting P21-activated kinase (PAK) by competing with ATP in the ATP binding site. This effect leads to an inhibition of vimentin phosphorylation, which is an important component of EMT [46]. This study was corroborated by others [73]. Moreover, in human pancreatic cancer cells, OSU inhibits PDK1 and AKT phosphorylation, thereby decreasing tumor invasion [32]. Finally, although the impact of DMC and OSU on angiogenesis is poorly known, one study has reported an ability of DMC to inhibit angiogenesis by inhibiting the secretion of the angiogenic factor ET-1 (Endothelin-1) [74]. Together, these data suggest that both molecules may reduce cancer cells dissemination.

### 3.5. Other Potential Mechanisms?

**Figure 3:**
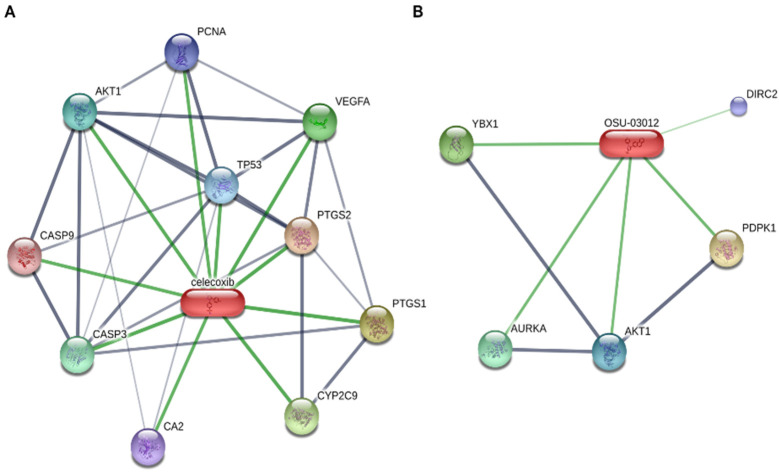
Proteins bound by celecoxib (**A**) or OSU-03012 (**B**). Data were retrieved from STITCH database (www.stitch.embl.de accessed on 29 April 2021).

The anti-tumoral effects of DMC and OSU is not strictly limited to the mechanisms described above. An analysis of the network of proteins bound by celecoxib and OSU (STITCH database: Figure 3) reveals that despite their structural similarities, these compounds regulate a different network of proteins. Although some of them were already discussed in the above paragraphs (e.g., AKT1, AURKA), other proteins, such as DIRC2 or YBX1 are also potentially bound/regulated by OSU-03012. Although the inhibitory activity of OSU on YBX1 was validated in the context of endometriosis [75], the impact of such interaction on cancer-related processes was never studied, despite their altered expression in several cancers [76,77,78]. Finally, the impact of DMC or OSU on immune cells remains to be determined. To date, only one study has shown that DMC increases the level of CD8^+^ T cells in a xenograft model of hepatitis B virus X (HBx) positive hepatoma [79]. Moreover, DMC reduces the expression of PD-L1 and CD163 in tumors, thus suggesting that DMC favors the immune antitumoral response [79]. Accordingly, combination of DMC with atezolizumab (anti-PD-L1 antibody) synergistically inhibit the PD-1/PD-L1 pathway.

## 4. Non-Active COX-2 Inhibitors in Combination with Other Therapeutic Approaches

Cancers are treated by surgery, chemotherapy, radiotherapy, photodynamic therapy, or combined therapeutics strategies, depending on the kind of cancer, the location of the tumors, the stage of the disease, etc. These therapies are also associated to severe side effects (e.g., sterility, immunodepression, anemia, alopecia, etc.). Therefore, combining existing therapeutic approaches with novel and efficient molecules can give synergistic or additive effects and thus may improve patients’ outcome and reduce adverse side effects. The combination of DMC or OSU with existing therapeutic approaches has been studied in various cancers and suggests promising therapeutic perspectives (Table 2).

### 4.1. DMC and OSU-03012 in Combination with Chemotherapy

The development of chemoresistance is a major cause of tumor recurrence and cancer-related mortality. Among the mechanisms involved, the overexpression of anti-apoptotic proteins such as IAPs (inhibitors of apoptosis) or BCL-2 family members, allow cancer cells to escape cell death. The ability of DMC and OSU to downregulate the expression of these anti-apoptotic proteins (e.g., Mcl-1, Bcl-2) may therefore represent an appealing strategy to re-sensitize cancer cells to chemotherapeutic agents. Only a few studies have been conducted to determine the efficiency of such combinations. OSU was identified in a drug-screening study aimed at identifying molecules that could overcome cisplatin resistance in ovarian cancer [80]. In particular, OSU could re-sensitize cisplatin resistant A2780 cells to cisplatin at concentrations below 20 μM. A synergistic effect between DMC and Perillyl alcohol, a monoterpene used for the treatment of systemic cancer was also observed in temozolomide-sensitive and -resistant glioma cells. This effect was associated to an ability of both drugs to trigger ER stress in cancer cells [80]. Together, these studies suggest a beneficial effect of DMC/OSU on chemotherapy. However, in one of our previous studies, the combination of DMC with different chemotherapeutic agents such as etoposide, did not give any beneficial effects [45], thus suggesting a cancer type-dependent benefit.

### 4.2. DMC and OSU-03012 in Combination with Radiotherapy

Radiotherapy is commonly used for the treatment of solid tumors such as colon, lung, breast, and prostate cancers [81]. The anti-tumoral properties of ionizing radiation are associated with DNA damage such as DNA double strand breaks, thereby triggering apoptosis through activation of tumor suppressor genes (e.g., p53, p63, p73). Some cancers, like melanoma are considered radio-resistant and thus combination therapy may improve the anti-tumoral response. Currently, very few studies are available regarding the combination of OSU or DMC with radiotherapy. OSU can sensitize glioblastoma cancer cells [82] and colorectal cancer cell lines (HCT-116) [83] to radiotherapy-induced apoptosis. Moreover, glioblastoma cells appeared to be more sensitive to OSU than non-transformed astrocytes, thus indicating that this molecule may preferentially affect cancer cells. Mechanistically, this effect has been associated to an induction of ER-stress and is PDK1 independent.

### 4.3. DMC and OSU-03012 in Combination with Photodynamic Therapy

Photodynamic therapy (PDT) refers to a therapeutic approach using a tumor localizing non-toxic photosensitive compound (called a photosensitizer), which become toxic upon excitation by exposure to a specific light [84]. Although several studies have shown that COX-2 inhibitors (i.e., celecoxib) could improve PDT efficiency in various cancer models [85], the combination of PDT with DMC or OSU has been poorly investigated. So far, only one study has demonstrated that DMC could improve PDT in a model of mouse mammary carcinoma (BA cells), using Photofrin^®^ as a photosensitizer [86]. This effect was associated to the downregulation of Survivin expression and an ER stress response [86]. Moreover, the capacity of DMC to improve PDT efficiency may serve for the treatment of several cancers for which Photofrin^®^, has been approved by the FDA (e.g., non-small cell lung cancer, esophagus cancer).

### 4.4. Other Potential Combinations

Targeted therapy. Few studies indicate that OSU or DMC can bring a beneficial effect in combination with targeted therapy. In one study, OSU could overcome resistance to imatinib mesylate (Gleevec), a potent inhibitor of BCR/Abl used for the treatment of CML [87]. This effect is mediated by an inhibition of the PI3K/AKT pathway at attainable therapeutic concentrations of imatinib (less than 5 µM). Interestingly this effect has been observed in TIB-196 myeloma cells but with a different mechanism, involving phospho-STAT3 downregulation and an increase of phospho-AMPK (Thr172) [88]. Similarly, combination of DMC and imatinib at half dose of the IC50 in colorectal cancer cells (HT-29) [89] gave a comparable effect to full dose monotherapy. This suggests that combining these molecules may reduce side effects associated to high doses of imatinib [90]. Similar beneficial effects of OSU have been reported with lapatinib, another tyrosine kinase inhibitor (ErbB1/ErbB2 inhibitor), in breast cancer [91] and glioblastoma [42]. In breast cancer cell lines (MDA-MB-231 cell line), this synergistic effect is mediated by the phosphorylation of eif2α (serine 51) through a decreased of the expression of Nck1 (NCK Adaptor Protein 1), a potent inhibitor of eif2α phosphorylation [91].

Immunotherapy. The combination of DMC or OSU with therapeutic antibodies has been poorly investigated. So far one study has shown that OSU-03012 can sensitize HER2-expressing breast cancer cells to trastuzumab [92]. This effect is associated to an impairment of PDK-1/AKT signaling and was not observed in HER2-negative cells (MDA-MB-231 cells). Finally, the combination of DMC with atezolizumab (an anti-PD-L1 antibody) synergistically inhibits the PD-1/PD-L1 pathway in HBV positive hepatoma tumors [79].

BH3 mimetics. Other combinations have been suggested in gastric cancer, such as DMC and ABT-737, a potent BH3 mimetic inhibiting BCL-2, BCL-xL and BCL-w activity [54]. This combination triggers a strong ER stress response and an induction of apoptosis through AIF (Apoptosis-Inducing Factor) activation in AGS and HGC-27 cells. This potential combination might be of interest for other cancers, where ABT-737 display anti-cancerous properties [93,94,95]. Importantly, the ability of DMC to impede MCL-1 expression in some cancers (e.g., leukemia) [45] may represent a promising approach to overcome resistance to ABT-737 [96,97]. Finally, a synergistic effect was observed in GBM with OSU in combination with a phosphodiesterase 5 (PDE5) inhibitor (e.g., sildenafil) [61].

More studies are required to evaluate the pertinence of such combinations, in particular, using in vivo approaches. Moreover, the combination of DMC/OSU with other BH3 mimetics, such as ABT-199 (venetoclax), Bcl-xL-specific A-1331852 or S63845, an MCL-1 specific inhibitor [98] has not been tested yet.

**Table 2:** Combinations between OSU or DMC with other anti-cancerous approaches.

Combinations	Cancer Type	Models Used	Mechanisms Behind a Better Response	References
OSU-03012/ Cisplatin	Ovarian Cancer	Cisplatin resistant A2780 cells	Increased apoptosis	[80]
DMC/ Perillyl Alcohol	Glioma	Temozolomide-sensitive and -resistant glioma cells	ER stress induction	[99]
OSU-03012/ Radiotherapy	Glioblastoma	Glioblastoma cancer cells	ER stress induction	[82]
	CRC	CRC cells: HCT-116	ER stress induction	[83]
DMC/PDT (Photofrin)	Breast Cancer	BA cells	ER stress induction	[100]
			Survivin downregulation	
DMC/ Chloroquin/ Nelfinavir	Breast Cancer	MDA-MB-231, MDA-MB-468	PARP, Caspase 7 and -3 cleavage	[30]
			Aggravation of ER stress	
			Autophagy induction	
OSU-03012/ Imatinib mesylate	CML	(Bcr)-Abl mutant cell lines: Ba/F3p210(E255K) Ba/F3p210(T315I)	PI3K/AKT pathway inhibition	[87]
	Myeloma	TIB-196 myeloma cells	Phospho-STAT3 downregulation	[88]
			Increased phospho-AMPK (Thr172)	
DMC/ Imatinib mesylate	CRC	HT-29 cells	Undetermined	[89]
OSU-03012/ Lapatinib	Breast Cancer	MDA-MB-231 cell line	Increased Eif2α phosphorylation Nck1downregulation	[91]
	Glioblastoma	GBM5,6, 12, 14 cells	Inhibition of multiple ERBB receptors	[42]
OSU-03012/ Trastuzumab	Breast Cancer	HER2-expressing breast cancer cells	Impairment of PDK-1/AKT signaling	[92]
DMC/ Trastuzumab	HCC	HBV positive hepatoma	Inhibition of PD-1/PD-L1 pathway	[79].
DMC/ABT-737	Gastric Cancer	AGS and HGC-27 cells	ER stress induction	[54]
OSU/Sildenafil	Glioblastoma	GBM5,6, 12, 14 cells	Death receptor signaling ER stress response	[61]

## 5. Are Non-Active COX-2 Inhibitors Devoid of Adverse Side Effects?

So far, no adverse-side effects have been clearly reported for these compounds, but most studies have been conducted in vitro. However, in 2009, a phase I clinical study was conducted for OSU-03012 with patients having advanced or recurrent solid tumors or lymphoma (Clinicaltrials.gov: NCT00978523, https://ascopubs.org/doi/abs/10.1200/jco.2013.31.15_suppl.2608 accessed on 29 April 2021) and OSU-03012 received an FDA-investigational new drug approval for cancer treatment. Although the results have not been posted yet, the authors indicate that the recommended phase II dose is 800 mg twice daily. Over this dose, signs of fatigue, dizziness, nausea, and rash were detected in some patients. Moreover, the study reports a high variability of the pharmacokinetic after a single dose, likely due to the dissolution of the formulation in the stomach. Surprisingly, in mice, oral administration of 200 mg/kg of OSU-03012 is well tolerated for 28 days and leads to a peak serum concentration >20 uM [101]. It should be noted that OSU-03012 is highly cytotoxic with an induction of cell death >50% from 5 μM in human monocyte-derived macrophages [102]. Similarly, our previous work demonstrated that DMC is toxic in PBMCs and in zebrafish embryos with >20 μM [45]. Moreover, OSU is likely hydrophobic, and its cellular internalization is poor [102]. Accordingly, other formulation such as polymeric microparticles (e.g., Acetalated Dextran) has been recently suggested to limit the toxicity and improve the delivery of this molecule [103]. The impact of these molecules in physiological processes and on the survival of healthy cells is also poorly known. Indeed, the ability of these molecules to impair the PI3K signaling may have detrimental effects on glucose/lipid metabolism by interfering with insulin signaling. In drosophila, DMC increases lifespan, improves the gut barrier, fecundity in females, and physical activity [104]. Part of these beneficial effects are associated to the ability of DMC to reduce AKT phosphorylation (T308) [104]. DMC can also inhibit PGE_2_ synthesis by blocking EGR1 expression, which activates mPGES-1 (microsomal prostaglandin E2 synthase) promoter [105,106]. Considering the important role of PGE_2_ in gastrointestinal mucosa renewal, it is therefore expected that DMC may lead to similar gastrointestinal side effects of celecoxib. The impact of OSU and DMC on cardiac function is also currently unclear. OSU induces cell swelling and reduces action potential duration of cardiomyocytes [107], while DMC prevents cardiomyocytes hypertrophy induced by isoprenaline in mice [108]. DMC also improves cardiac function (improvement of left ventricular systole) and prevents hypertrophic cardiac remodeling in a mouse model of inherited dilated cardiomyopathy (DCM) [109]. DMC also prevents pressure-induced cardiac remodeling by activating GSK3β [110]. DMC has a beneficial effect on the kidneys, as evidenced by a study of Yamamoto et al., showing that DMC prevents cardiac remodeling and kidney injury (reduced podocytes injury, glomerulosclerosis and fibrosis) in mice treated with angiotensin II and high salt [111]. These effects are mediated by an activation of GSK3β and the inhibition of the Wnt/β-catenin signaling in both organs. Recently, celecoxib and DMC were shown to promote calcification of the porcine aortic valve as evidenced in aortic valve interstital cells (PAVICs) cultured in osteogenic media. [112]. This effect was associated to an induction of RUNX2 and is glucocorticoid-dependent.

Together these findings indicate that although these drugs possess potent anti-cancerous properties, their potential side effects are currently largely unknown, thus suggesting cautions regarding their use in clinical practice. Intense efforts are still required to fulfill these gaps, especially by using in vivo models.

## 6. Conclusions

Non-active COX-2 inhibitors possess potent and stronger antitumor properties than celecoxib. Moreover, these molecules enhance the efficiency of existing therapies, thus suggesting interesting combinations approaches in clinical practice. Mechanistically, these drugs appear not only as potent ER stress inducers/aggravators but also inhibitors of the PI3K/AKT and of the Wnt/β-catenin signaling. Therefore, these drugs may represent appealing therapeutic molecules, as most AKT inhibitors display a poor bioavailability, specificity, and a poor activity due to the development of tumor resistance. However, the mechanisms of these drugs have not been completely depicted and the potential side effects need further investigations. Of note, other analogs of OSU have been recently developed, such as P12-3 and P12-34 [23], which display higher antiviral properties than OSU but have not been tested yet on cancer models. Therefore, a thorough assessment of the antitumoral effects of all these molecules and also their potential side effects using in vivo models is tremendously needed. Nevertheless, the potent antitumor properties of these molecules could be exploited for the design of new and effective therapeutic approaches for cancers for which very few and poorly efficient therapeutic options exist. Finally, these molecules may also represent novel weapons against other disorders, as evidenced for OSU, which inhibits SARS-CoV-2 replication [26].

## Figures and Tables

**Table 1:** Impact of DMC and OSU-03012 in cancers.

Cancers	Molecule	Processes	Targets	Expression	Models	References
Colorectal Cancer	DMC	Induces Apoptosis	Survivin	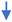	HCT-116 cells	[28]
		Inhibits cell cycle	Cyclin D1	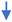		
Breast Cancer	OSU	Induces Apoptosis	p-AKT	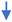	MDA-MB-453, MCF-7, T47D, MDA-MB-231 and HBL100	[29]
			PARP cleavage	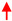		
	DMC + Chloroquine and Nelfinavir	Induces Apoptosis	PARP cleavage	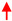	MDA-MB-231, MDA-MB-468, Hs578t, T47D and MCF-7	[30]
		Induces ER stress	GRP78, CHOP	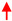		
CLL	OSU	Induces Intrinsic Apoptosis	Cleaved caspase 3/9 and PARP	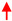	PBMCs from patients with CLL	[31]
Pancreatic cancer	OSU	Inhibits Migration/ Invasion	PDK1/p-AKT	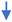	sPC-1, BxPC-3, Mia- PaCa 2, and PANC-1	[32]
Prostate cancer	OSU	Inhibits Cell cycle Induces Apoptosis	PDK1/AKT signaling	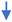	PC-3 cells	[33]
			PDK1 expression	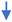		
Hepatocellular carcinoma	DMC	Increases Autophagy	LC3II	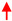	SNU-354, SNU-423, SNU-449 and SNU-475 cells	[34]
			p-AMPK	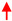		
			p-mTOR	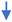		
		Induces ER stress	CHOP	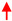		
		Induces extrinsic Apoptosis	c-FLIP	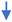		
			DR5	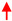		
	OSU	Induces Autophagy	ROS	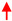	Huh7, Hep3B and HepG2 cells	[35]
			ATG5 expression	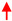		
		Inhibits Growth, Cell cycle	Undetermined	-		
Lung cancer	DMC	Induces apoptosis	CHOP	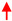	Human Non-Small-Cell Lung Cancer cells	[36]
			DR5	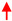		
			c-FLIP	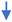		
Glioblastoma	DMC	Induces Apoptosis	Survivin	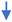	A172, U87, SNB75 and U251 cells	[37]
			Cleaved caspase 3 and PARP	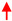		
		Inhibits Cell cycle Induces apoptosis	p21	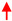	LN229, U87MG, A172, and U251 cells	[38]
			CIP2A/PP2A/AKT	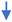		
		Induces ER stress	GRP78	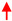		
			CHOP	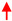		
	OSU	Induces ER stress	GRP78/Bip	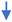	GBM12, GBM14 GBM6-luciferase cells (xenograft in mice)	[39]
			p-PERK	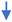		
		Increased Autophagy	LC3II	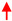	U-373 MG and U-87 MG	[40]
		Induces Apoptosis	MCL1	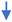	Primary GBM cells, GBM6, 12, 14 cells	[41]
			BcLxL	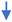		
			PI3K/AKT ERK1/2 (in combination with lapatinib)	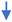	GBM5,6,12 and 14	[42]
				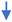		
Human oesophageal carcinoma	OSU	Induces Apoptosis	BAX	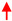	Eca-109, TE-1, and TE-11 cells	[43]
			p-p53	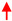		
			Cleaved caspase 3	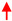		
			Cleaved caspase 9	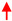		
			Cytochrome c release	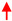		
Burkitt Lymphoma	DMC	Inhibits Cell cycle	Cyclin A/B	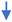	Raji, Ramos and A6876 cells	[44]
			P27Kip1	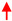		
Leukemia	DMC	Arrest of Cell cycle (G1/S)	c-myc	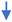	U937, K562, Raji, Hel cells	[45]
		Induces apoptosis	Survivin	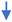		
Thyroid cancer	OSU	Inhibits cell cycle (S phase accumulation)	p-AKT	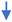	NPA (papillary) WRO (follicular) ARO (anaplastic) cells	[46]
		Induces apoptosis	Undetermined	-		
		Migration/Invasion	PAK	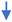		

## Data Availability

Proteins bound by celecoxib or OSU-03012 (Figure 3) were retrieved from STITCH database (www.stitch.embl.de accessed on 29 April 2021).

## Abbreviations

AIF: Apoptosis-Inducing Factor. AMPK: AMP-activated protein kinase. APAF1: *Apoptotic* peptidase activating factor 1. ATF6: Activating transcription factor 6. ATG: autophagy-related protein. BAG2: BCL2 associated athanogene 2. BAK: Bcl-2 homologous antagonist killer. BAX: Bcl-2–associated X protein. Bcl-2: B cell lymphoma 2. Bcl-xL: B cell lymphoma extra-large. BH3: Bcl-2 homology domain-3. c-FLIP: cellular FLICE (FADD-like IL-1β-converting enzyme)-inhibitory protein. CHOP: C/EBP Homologous Protein. CLL: Chronic lymphocytic leukemia. COX: cyclooxygenase. CPC: chromosomal passenger complex. DHOH: Dihydroorodate deshydrogenase. DMC: 2,5-dimethyl celecoxib. DR5: death receptor 5. ER: endoplasmic reticulum. ERAD: Endoplasmic Reticulum-Associated Protein Degradation. FasL: Fas Ligand. GBM: glioblastoma multiform. GRP78: glucose-regulated protein 78. GSK: glycogen synthase kinase. HSP27: Heat shock protein 27. HSP75: Heat shock protein 75. IAPs: Inhibitors of Apoptosis. Il: Interleukin. IRE1: Inositol-Requiring Enzyme 1. LC3: Microtubule Associated Protein 1 Light Chain 3. LO: lipoxygenase. LTB_4_: leucotriene B4. Mcl-1: myeloid cell leukemia 1. MDA7: melanoma differentiation associated gene-7. MiR: microRNA. MMP: Matrix Metalloproteinases. mPGES-1: microsomal prostaglandin E2 synthase. mTORC1: mammalian target of rapamycin complex 1. NFkB: nuclear factor kappa B. NSAIDs: non-steroidal anti-inflammatory drugs. NSCL: Non-Small Cell Lung Cancer. OSU: OSU-03012. PAK1: p21-Activated Kinase 1. PARP: poly(ADP-ribose) polymerase. PDE_5_: *phosphodiesterase 5*. PDK1: Pyruvate dehydrogenase kinase 1. PDT: Photodynamic therapy. PERK: protein kinase R (PKR)-like endoplasmatic reticulum kinase. PGG_2_: prostaglandin G2. PGH_2_: prostaglandin H2. PGI_2_: prostaglandin I_2_. PGIS: prostacycline synthase. PI3CA: phosphatidylinositol 4,5 bisphosphate 3 kinase catalytic subunit α. PI3K: Phosphatidylinositol-3-Kinase. ROS: reactive oxygen species. SERCA: sarco/endoplasmic reticulum Ca^2+^-ATPase. STAT3: Signal transducer and activator of transcription 3. TCF7L2: T-cell factor-like 2. TCRP1: Tongue cancer resistance-related protein 1. TL: Toll like receptor. TNF: Tumor necrosis factor. TRAIL: TNF-related apoptosis-inducing ligand. TXA_2_: Thromboxane A2. ULK-1: Unc-51 like autophagy activating kinase 1. UPR: unfolded protein. XBP1: X-box binding protein 1. XIAP: X-linked inhibitor of apoptosis protein
